# Long-Term Results and Prognostic Factors of Gastric Cancer Patients with Microscopic Peritoneal Carcinomatosis

**DOI:** 10.1371/journal.pone.0037284

**Published:** 2012-05-16

**Authors:** Xiaowen Liu, Hong Cai, Weiqi Sheng, Yanong Wang

**Affiliations:** 1 Department of Gastric Cancer and Soft Tissue Sarcoma, Fudan University Shanghai Cancer Center, Shanghai, China; 2 Department of Pathology, Fudan University Shanghai Cancer Center, Shanghai, China; 3 Department of Oncology, Shanghai Medical College, Fudan University, Shanghai, China; The Chinese University of Hong Kong, Hong Kong

## Abstract

**Background:**

Clinical significance of microscopic peritoneal carcinomatosis remained unclear. The aim of this study was to evaluate the prognostic value of microscopic peritoneal carcinomatosis in gastric cancer.

**Methods:**

From 1996 to 2007, 4426 patients underwent gastrectomy for gastric cancer at Fudan University Shanghai Cancer Center. The clinical and pathological data were reviewed to identify patients with microscopic peritoneal carcinomatosis (group 1). The clinicopathological features and prognosis were examined. Additionally, 242 stage-matched gastric cancer patients without microscopic peritoneal carcinomatosis (group 2) and 118 with macroscopic peritoneal carcinomatosis (group 3) were selected as control groups.

**Results:**

Microscopic peritoneal carcinomatosis was found in 121 patients. There were 85 males and 36 females (2.36:1). There was a higher incidence rate of large size tumor (≥5 cm) (*P* = 0.045), Borrmann IV (*P* = 0.000), and serosal invasion (*P* = 0.000) in gastric cancer with microscopic peritoneal carcinomatosis compared with the control group. The 5-year survival rate of gastric cancer with microscopic peritoneal carcinomatosis was 24%, significantly poorer than that of the stage-matched control group but better than that of patients with macroscopic peritoneal carcinomatosis. The independent prognostic factors identified included pathological stage and operative curability.

**Conclusions:**

The presence of microscopic peritoneal carcinomatosis was associated with worse prognosis for gastric cancer, but curative surgery showed potential to improve prognosis.

## Introduction

Although the incidence of gastric cancer has been substantially declining for several decades, it remained the fourth most common cancer and the second most frequent cause of cancer death worldwide [1], [2]. It was very important to predict precisely the risk of poor prognosis in order to maximize the therapeutic effect and to minimize the adverse effects in the treatment of cancer patients. Among the prognostic factors now available for gastric cancer, the most precise and useful prognostic factor was the UICC TNM (tumor, lymph node, and metastasis) staging stage. Peritoneal metastasis was considered to be one of the metastasis, and was one of the most common types of spread and the causes of death [3]. Peritoneal metastasis of gastric cancer was considered to be operation contraindication and the most difficult type for treatment [4]. The peritoneal metastasis was mainly classified classified as macroscopic peritoneal carcinomatosis (overt peritoneal dissemination) and positive peritoneal lavage cytology, and their prognostic value has been extensively investigated in gastric cancer. However, tumor nodules were occasionally found in the peritoneal of gastric cancer patients by histopathological examination. We defined this kind of peritoneal dissemination as microscopic peritoneal carcinomatosis compared with macroscopic peritoneal carcinomatosis. There was currently no evidence as to the clinical significance of microscopic peritoneal carcinomatosis in gastric cancer.

The objectives of this study were to investigate the clinical significance of microscopic peritoneal carcinomatosis and to assess the impact of microscopic peritoneal carcinomatosis on survival related to clinicopathological characteristics in patients with resectable gastric cancer.

## Materials and Methods

From January 1996 to December 2007, 4426 patients with histologically confirmed primary gastric adenocarcinoma underwent gastrectomy at the Department of Surgery in Fudan University Shanghai Cancer Center. The electronic records of these patients were reviewed, and patients with microscopic peritoneal carcinomatosis were included in this study. Microscopic peritoneal carcinomatosis was defined as the nodules without any evidence of lymph node tissue or lymph node architecture, and cannot be found intraoperatively. In this study, the location of resected peritoneum included greater omentum, lesser omentum, and transverse mesocolon according to guideline of gastric cancer therapy.

Additionally, 242 stage-matched gastric cancer patients without peritoneal dissemination (group 2) and 118 with macroscopic peritoneal carcinomatosis (group 3) were selected as control. All patients of group 3 received gastrectomy. Before operations, all patients were routinely performed upper gastrointestinal barium-meal, endoscopic examination, abdominal ultrasound, and computed tomographic scan. Staging was performed according to the American Joint Committee on Cancer (AJCC) TNM Staging Classification for Carcinoma of the Stomach (7th edition, 2010). Data were retrieved from patients' operative and pathological reports, and follow-up data were obtained by phone, letter, and the out-patient clinical database. The written informed consent had been obtained from all the patients, and this study was approved by the Ethical Committee of Shanghai Cancer Center of Fudan University. The study was retrospective.

### Follow-Up

Postoperative follow-up included physical examination, imaging examination, and laboratory examination every three months for the first two years at the outpatient, every six months for the next 3 years, and after 5 years every 12 months for life. Overall survival, which was used as a measure of prognosis, was defined as time from operation to death or last follow-up.

### Statistical analysis

The clinicopathological comparisons between patients with microscopic peritoneal carcinomatosis and control groups were evaluated by Fisher exact test. Five-year survival rates were calculated by Kaplan-Meier method, and differences between survival curves were examined with Log-rank test. The accepted level of significance was *P*<0.05. Statistical analyses and graphics were performed with the SPSS 13.0 statistical package (SPSS, Inc., Chicago, IL).

## Results

### Clinicopathological characteristics

There were 85 males and 36 females (2.36:1) with a mean age of 59 years. According to histological type, well-differentiated were observed in 2 (1.7%) patients, moderately-differentiated in 28 (23.1%) patients, and poorly-differentiated in remaining 91 (75.2%) patients. According to Borrmann type, 9 (7.4%) type I, 1 (0.8%) type II, 100 (82.6%) type III, 11 (9.1%) type IV. Of 121 patients, 37 (30.6%) had tumors located in the upper third, 31 (25.6%) had tumors in the middle third, 45 (37.2%) had tumors in the lower third of the stomach, and 8 (6.6%) had tumors occupied two-thirds or more of stomach. Lymph node metastasis was observed in 101 patients, the total metastasis rate was 83.5%. The distribution of pathological stage was as follows: 4 (3.3%) patients belonged to stage IB, 18 (14.9%) IIB, 19 (15.7%) IIIA, 33 (27.3%) IIIB, 88 (17.6%) IIIA, 47 (38.8%) IIIC.

Clinicopathologic characteristics of patients with microscopic peritoneal carcinomatosis (group 1) were compared with that of gastric cancer without microscopic peritoneal carcinomatosis (group 2) and gastric cancer with macroscopic peritoneal carcinomatosis (group 3). Results showed that sex, tumor location, histology type, and surgical properties were similar between group 1 and group 2. There was a higher incidence rate of older patients (≥60) (*P* = 0.012), large size tumor (≥5 cm) (*P* = 0.045), Borrmann IV (*P* = 0.000), and serosal invasion (*P* = 0.000) in group 1(Table 1). There were significant differences of age and tumor location between group 1 and group 3 (Table 2).

**Table 1:** Comparison of Clinicopathological Features Between gastric cancer patients with microscopic peritoneal carcinomatosis (group 1) and gastric cancer without microscopic peritoneal carcinomatosis (group 2).

Variables	Group 1	Group 2	*P*
Sex (M/F)	85/36	165/77	0.689
Age (≥60/<60)	70/51	106/136	0.012
Histology type (P/M/W)*	91/28/2	180/61/1	0.408
Operation curability (yes/no)	102/19	206/36	0.836
Tumor size (≥5/<5)	71/50	115/127	0.045
Borrmann type IV (yes/no)	11/110	1/241	0.000
Serosal invasion (yes/no)	114/7	122/120	0.000
Tumor location (C/M/A and two or more)#	37/31/45/8	80/42/106/14	0.280

* Poorly differentiated/moderately differentiated/well differentiated.

# Corpus/middle/antrum.

**Table 2:** Comparison of Clinicopathological Features Between gastric cancer patients with microscopic peritoneal carcinomatosis (group 1) and gastric cancer with macroscopic peritoneal carcinomatosis (group 3).

Variables	Group 1	Group 3	*P*
Sex (M/F)	85/36	74/44	0.217
Age (≥60/<60)	70/51	49/69	0.012
Histology type (P/M/W)*	91/28/2	97/18/3	0.263
Tumor size (≥5/<5)	71/50	69/49	0.975
Borrmann type IV (yes/no)	11/110	19/99	0.102
Serosal invasion (yes/no)	114/7	103/15	0.064
Tumor location (C/M/A and two or more)#	37/31/45/8	16/35/45/22	0.002

* Poorly differentiated/moderately differentiated/well differentiated.

# Corpus/middle/antrum.

### Tumor nodules features

Microscopic peritoneal carcinomatosis was located in the different location of peritoneal. 83 (68.6%) had microscopic peritoneal carcinomatosis located in the serosal surface of stomach, 27 (22.3%) had it in greater omentum, 2 (1.7%) had it in lesser omentum, 4 (3.3%) in transverse mesocolon, and 5 (4.1%) had it occupied in two or more parts. Single microscopic peritoneal carcinomatosis was found in 62 patients, two or more microscopic peritoneal carcinomatoses were found in other patients. The total number of microscopic peritoneal carcinomatosis was 249 (mean 2.0 and median 1.0 per patient).

### Prognosis

**Figure 1:**
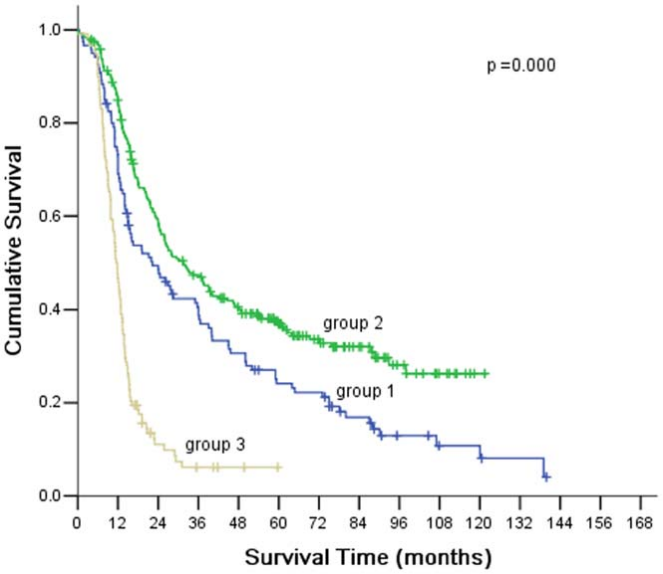
There were significant differences in the survival among three groups (patients with microscopic peritoneal carcinomatosis: group 1; without microscopic peritoneal carcinomatosis: group 2; with macroscopic peritoneal carcinomatosis: group 3).

The 1-, 3-, 5-yr survival rates of the gastric cancer with microscopic peritoneal carcinomatosis were 73%, 41%, and 24%, respectively, the 1-, 3-, 5-yr survival rates of the gastric cancer without microscopic peritoneal carcinomatosis were 85%, 47%, and 37%, respectively, and that of gastric cancer with macroscopic peritoneal carcinomatosis were 47%, 6%, and 6%. These differences were statistically significant among three groups (*P* = 0.000) (Figure 1). The significant prognostic factors of the gastric cancer with microscopic peritoneal carcinomatosis included: the number of tumor nodules, serosal invasion, operative curability, lymph node metastasis, and pathological stage. The independent prognostic factors included: pathological stage and operative curability (Table 3). The 5-year survival rate was 28% in patients who underwent curative surgery.

**Table 3:** Multivariate analysis on factors in influencing survival.

Variable	χ^2^	*P* value	Hazard ratio	95% CI
Sex	0.297	0.586	1.131	0.726–1.762
Age	0.325	0.569	1.130	0.742–1.723
Number of MPC*	2.068	0.150	1.351	0.897–2.037
Serosal invasion	0.173	0.678	0.762	0.211–2.750
The status of lymph node	0.010	0.921	1.053	0.381–2.912
Pathological stage	11.474	0.001	1.780	1.275–2.484
Operation curability	27.844	0.000	0.212	0.119–0.378

* MPC microscopic peritoneal carcinomatosis.

## Discussion

Gastric cancer was one of the most common malignancies around the world. Although the prognosis of patients with gastric cancer has improved as a result the availability of diagnostic techniques and better therapy strategy, gastric cancer was still the second leading cause of cancer related deaths [2]. The dismal prognosis of gastric cancer was due principally to the frequent metastasis. The most frequent type of metastasis in gastric cancer was peritoneal carcinomatosis (PC) [5]. In the Japanese Rules of Gastric Cancer, PC was classified into five categories: P0/Cy0, P0/Cy1, P1, P2 and P3. P0/Cy0 denoted no macroscopic disease and negative peritoneal wash cytology; P0/Cy1 meant no macroscopic PC but positive peritoneal wash cytology; P1 denoted PC in the upper abdomen above the transverse colon; P2 meant several countable PC in the peritoneal cavity; and P3 meant numerous PC in the peritoneal cavity. However, we found that some tumor nodules were occasionally found in the peritoneal of gastric cancer patients by histopathological examination. This kind of peritoneal carcinomatosis entitled as microscopic peritoneal carcinomatosis was not included into the gastric cancer staging system. The prognostic significance of microscopic peritoneal carcinomatosis in gastric cancer was still unclear. There have been no prior reports in the literature investigating this type of peritoneal dissemination, and therefore the incidence of microscopic peritoneal carcinomatosis remained unknown. In this study, 121 patients were classified as having microscopic peritoneal carcinomatosis based on the histologic examination, the incidence was 2.7%, which was lower than that of the macroscopic peritoneal carcinomatosis or positive peritoneal lavage cytology [6], [7].

In this study, we found that there was a higher incidence of large size tumor, Borrmann IV, and serosal invasion in gastric cancer patients with microscopic peritoneal carcinomatosis than that of patients without microscopic peritoneal carcinomatosis. In one of the earlier study, Kostić et al. [8] showed that a tumor diameter >5 cm, tumor invasion of serosa, histopathological stage of the disease III and IV, and macroscopically visible metastases were the most important risk factors for detection of free cancer cells in patients surgically treated for gastric adenocarcinoma. The exact mechanism that was contributing to microscopic peritoneal carcinomatosis was still not clear. Yonemura [9] suggested that peritoneal dissemination was associated with lymphatic orifices of peritoneal surfaces. The orifices were referred to as the lymphatic stomata, and connected with the subperitoneal lymphatic channel and milky spots. Milky spots were the minute organelles, which contained lymphatic vessels, lymphocytes, and peritoneal macrophages. Intraperitoneal free cancer cells specifically deposited in the lymphatic stomata, and proliferated in the submesothelial lymphatic space. Additionally, they also found that milky spots distributed mainly on the greater omentum and pelvic peritoneum. According to this theory, we hypothesized that peritoneal cancer nodules should mainly distributed on the greater omentum and pelvic peritoneum. However, this was not the case. In current study, we found that most of the patients (68.6%) had microscopic peritoneal carcinomatosis located in the serosal surface of stomach, and only 22.3% in greater omentum. Therefore, it was possible that there were some other mechanisms which facilitated peritoneal dissemination.

It was well-known that the prognosis of gastric cancer patients with macroscopic peritoneal carcinomatosis or positive peritoneal lavage cytology was dismal. It was reported that the prognosis of patients with peritoneal carcinomatosis and ascites was very poor, with a median survival of 3–6 months and no long-term survivors [10], [11]. Saito et al. [12] reported that the 5-year survival rate of advance gastric cancer with intraperitoneal free cancer cells was 15.3%. Up to now, the prognosis and the clinicopathological characteristics related to the prognosis of gastric cancer patients with microscopic peritoneal carcinomatosis have not been identified. In the current study, the 5-yr survival rate of patients with microscopic peritoneal carcinomatosis was 24%, which was significantly poorer than that of the gastric cancer without microscopic peritoneal carcinomatosis, but better than that of gastric cancer with macroscopic peritoneal carcinomatosis. The independent prognostic factors included: pathological stage and operative curability. The 5-year survival rate was 28% in patients with curative surgery, and 60% in patients with stage I/II. For the 19 patients presenting with stage I/II who underwent curative surgery, the 5-year survival rate of these patients was 64%. Therefore, good survival rate can be expected in I/II stage patients with microscopic peritoneal carcinomatosis, who received curative gastrectomy.

Although we firstly reported the prognosis of patients with microscopic peritoneal carcinomatosis, there were some limitations to this study. First, this study was limited by its retrospective nature, and selection bias may have influenced survival data. Second, intraoperative chemotherapy was not incorporated into the analysis. Some studies have the efficacy of intraperitoneal chemotherapy in gastric cancer patients with peritoneal dissemination [13], [14]. Third, the number of patients was small and a larger number of patients would be required to confirm these results. Fourth, although all the patients received resection of peritoneum or biopsies of peritoneum, the part of resected peritoneum only included greater omentum, lesser omentum, and transverse mesocolon according to guideline of gastric cancer therapy. Therefore, the frequency of gastric cancer with microscopic peritoneal carcinomatosis might be higher than the suggested.

In conclusion, the prognosis of gastric cancer patients with microscopic peritoneal carcinomatosis was poorer than that of patients without microscopic peritoneal carcinomatosis. Radical surgery should be performed for early stage patients with microscopic peritoneal carcinomatosis in order to improve survival outcomes.

## References

[1] Shibata A, Parsonnet J, Schottenfeld D, Fraumeni JF, Stomach cancer (2006). Cancer epidemiology and prevention, 3rd edn.

[2] Parkin DM, Bray F, Ferlay J, Pisani P (2005). Global cancer statistics, 2002.. CA Cancer J Clin.

[3] Yamada E, Miyaishi S, Nakazato H (1980). The surgical treatment of cancer of the stomach.. Int Surg.

[4] Sadeghi B, Arvieux C, Glehen O, Beaujard AC, Rivoire M (2000). Peritoneal carcinomatosis from nongynecologic malignancies: Results of the EVOCAPE 1 multicentric prospective study.. Cancer.

[5] Saito H, Tsujitani S, Kondo A, Ikequchi M, Maeta M (1999). Expression of vascular endothelial growth factor correlates with hematogenous recurrence in gastric carcinoma.. Surgery.

[6] Oh CA, Bae JM, Oh SJ, Choi MG, Noh JH (2012). Long-term results and prognostic factors of gastric cancer patients with only positive peritoneal lavage cytology.. J Surg Oncol.

[7] Sarela AI, Miner TJ, Karpeh MS, Coit DF, Jaques DP (2006). Clinical outcomes with laparoscopic stage M1, unresected gastric adenocarcinoma.. Ann Surg.

[8] Kostić Z, Cuk V, Bokun R, Ignjatović D, Usaj-Knezević S (2006). Detection of free cancer cells in peritoneal cavity in patients surgically treated for gastric adenocarcinoma.. Vojnosanit Pregl.

[9] Yonemura Y, Yonemura Y (1998). Mechanisms of the formation of peritoneal dissemination.. Peritoneal dissemination.

[10] Sakata Y, Ohtsu A, Horikoshi N, Suqimachi K, Mitachi Y (1998). Late phase II study of novel oral fluoropyrimidine anticancer drug S-1 (1 M tegafur-0.4 M gimestat-1 M ostat potassium) in advanced gastric cancer patients.. Eur J Cancer.

[11] Koizumi W, Narahara H, Hara T, Takagane A, Akiya T (2008). S-1 plus cisplatin versus S-1 alone for first-line treatment of advanced gastric cancer (SPIRITS trial): a phase III trial.. Lancet Oncol.

[12] Saito H, Kihara K, Kuroda H, Matsunaga T, Tatebe S (2011). Surgical outcomes for gastric cancer patients with intraperitoneal free cancer cell, but no macroscopic peritoneal metastasis.. J Surg Oncol.

[13] Kuramoto M, Shimada S, Ikeshima S, Matsuo A, Yaqi Y (2009). Extensive intraoperative peritoneal lavage as a standard prophylactic strategy for peritoneal recurrence in patients with gastric carcinoma.. Ann Surg.

[14] Yang XJ, Huang CQ, Suo T, Mei LJ, Yang GL (2011). Cytoreductive surgery and hyperthermic intraperitoneal chemotherapy improves survival of patients with peritoneal carcinomatosis from gastric cancer: final results of a phase III randomized clinical trial.. Ann Surg Oncol.

